# Cardiotoxicity of verapamil in renal failure: a case report and review of the literature

**DOI:** 10.4076/1757-1626-2-6312

**Published:** 2009-09-01

**Authors:** Praveen P Jadhav, Suresh Bohra

**Affiliations:** Department of Medicine, Goulburn Valley HealthGraham Street, Shepparton, Vic 3130Australia

## Abstract

We present a case of a 76-year-old diabetic patient on verapamil with undiagnosed renal failure presenting with collapse and severe life threatening bradyarrhythmias. She responded well to inotropic support and calcium supplementation.

## Case presentation

**Figure 1.**
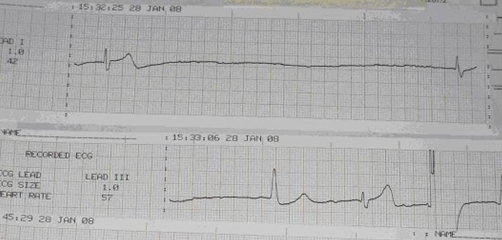
ECG prior to admission showing marked bradyarrhythmia.

A 76-year-old Anglo-Saxon Australian woman presented 15 minutes history of dizziness, lightheadedness, diaphoresis and collapse. According to the paramedics, her heart rate was 30 per minute, blood pressure was not recordable and heart sounds were feeble. She was given 1.2 mg atropine and an adrenaline infusion was started at the rate of 2 microgram/min. There was no history of chest pain, palpitations, shortness of breath, loss of consciousness, fall, seizures or injury. She was brought to the emergency department with oxygen and adrenaline infusion. The ECG rhythm strip prior to admission showed long pauses extending more than 8 seconds, and junctional and ventricular beats (Figure 1). On admission pulse was 50 per minute, small volume, blood pressure 86/50 mmHg, respiratory rate 14 per minute and had oxygen saturation of 98%. She was sweating and appeared sick. Systemic examination was unremarkable except basal crepitations posteriorly.

Further enquiry revealed that she had history of type II diabetes for about 20 years. This was controlled with regular treatment with gliclazide 30 mg OD. There was a history of hypertension for which she was taking verapamil 480 mg daily, and furosemide 20 mg OD. Thyroid nodule was removed more than 20 years ago, which was non cancerous and did not require any further treatment. She had an episode of paroxysmal atrial fibrillation 6 years ago, which did not recur. There was no history of ischemic heart disease, peripheral vascular disease or renal disease.

Differential diagnosis of ischemic episode, sick sinus syndrome and verapamil toxicity were considered. Treatment was continued with inotropes. Central line was inserted and transvenous cardiac pacemaker was not inserted as she was hemodynamically stable, though kept ready. Presuming it to be an ischemic episode aspirin, enoxaparin and atorvastatin was started.

Investigations revealed hemoglobin of 118 g/l, white cell count of 18.3/nL, neutrophils of 14.7/nL. Cardiac enzymes were normal (Trop T < 0.01). Serum creatinine was 200 µm/L (normal 55-110 µm/l), urea 18.8 mmol/l (normal 3.3-7.6 uM/L), calcium 2.36 mmol/l (normal 2.15-2.55 mmol/l), magnesium 1.12 mmol/l (normal 0.65-1.05 mmol/l), phosphate 1.57 mmol/L (normal 0.87-1.45 mmol/l) and other electrolytes were normal. Arterial blood gases showed pH 7.26, PO_2_ 29 mmHg, and PCO_2_ – 57 mmHg. Random blood sugar was 19.4 mmol/L. Thyroid function tests were normal. ECG showed a junctional rhythm with a rate of 60/minute. Echocardiography showed left ventricular hypertrophy and normal ejection fraction.

**Figure 2.**
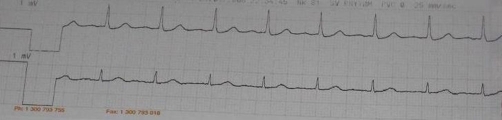
ECG after treatment demonstrating improvement following administration of inotropes and intravenous calcium.

Inotropes increased the heart rate but with junctional rhythm. Hypermagnesemia was reverted with 10 ml of 10% Calcium gluconate given intravenous over 10 minutes. Few minutes after the administration, the ECG changed to atrial fibrillation with a ventricular rate of 96/min (Figure 2).

She was transferred to intensive care unit; adrenaline infusion continued and later tapered off. Insulin (regular) infusion was started for blood glucose control. The patient was up and fit the next day. During the course of next 3 days, she went into rapid ventricular response with atrial fibrillation and had to be controlled with intravenous metoprolol. She remained in-patient for 4 days. Creatinine dropped from 200 to 128 µmol/l, urea from 18.8 to 12.7 mmol/L and magnesium from 1.12 to 0.88 mmol/l. She was discharged asymptomatic with rate controlled atrial fibrillation.

## Discussion

Verapamil is a commonly used calcium channel blocker for management of hypertension, supraventricular tachycardia, hypertrophic cardiomyopathy, angina pectoris, mitral stenosis, migraine prophylaxis. It is also used to treat hypertension associated with renal failure. Therapeutic recommended doses are up to 480 mg/day [1]. Though dose adjustment of verapamil has been suggested in renal failure, it is not adequately stressed. It blocks rapid influx of calcium into the cardiac myocytes of cardiac conduction system and smooth muscle vasculature resulting in decreased myocardial contractility, prolonged conduction time, and vascular relaxation. Earlier reports have suggested that slow release verapamil can cause acute toxic effects (slow cardiac rhythms and hypotension) in patients with chronic renal failure [2,3]. Death with 720 mg sustained release verapamil has also been reported [4]. Though our patient was on therapeutic doses, she was taking a dose in the higher range of the recommended.

The mechanism of cardiotoxicity in renal failure has been suggested to be due to accumulation of verapamil or its metabolites due to their reduced excretion [2]. However, the role of magnesium in causing its toxicity has not been studied.

Hypermagnesemia is commonly seen in renal failure [5]. Magnesium is an effective calcium channel blocker, which when elevated, results in bradyarrhythmias [6]. Its toxicity is linear and includes neuromuscular effects such as depressed deep tendon reflexes, somnolescence, muscle paralysis and quadriplegia. It causes hypocalcemia due to parathyroid suppression. The effects on heart include suppression of sinus node, delaying conduction through the atrioventricular node and hypotension. Increased levels can lead to prolongation of PR interval, increased QRS duration and an increase in QT interval. In terminal stages complete heart block and cardiac arrest can occur.

Since magnesium and verapamil both block the calcium channels in the cardiac muscle, their actions may be additive or synergistic. Hence, dose of verapamil bordering the upper level of recommended may be harmful in settings of hypermagnesemia. Conversely, lower than reported toxic levels of magnesium may produce toxicity in conjunction with verapamil. With no other likely diagnosis and no evidence of acute cardiac insult, this mechanism could have led to severe bradyarrhythmia in our patient despite ‘therapeutic’ doses of verapamil and mildly elevated magnesium levels. This is further supported by the temporal association of administration of injection calcium gluconate and the heart rate picking up within seconds in this patient. Though verapamil levels were not tested in this patient, it could have given an additional evidence for the diagnosis.

Therefore, we suggest that in patients with renal failure verapamil should be given in lower doses. For these patients, renal functions and magnesium levels should be monitored regularly. This suggestion is particularly important in hypertensive persons with diabetes who have a greater propensity for development of renal failure. In the event of development of cardiac toxicity in the form of bradyarrhythmias, calcium gluconate infusion should be the treatment of choice apart from inotropic support.

## Abbreviation

ECG: electrocardiogram
